# Rethinking Strategies for Multi-Metastatic Patients: A Comprehensive Retrospective Analysis on Open Posterior Fusion Versus Percutaneous Osteosynthesis in the Treatment of Vertebral Metastases

**DOI:** 10.3390/jcm13113343

**Published:** 2024-06-06

**Authors:** Laura Scaramuzzo, Andrea Perna, Calogero Velluto, Maria Ilaria Borruto, Franco Lucio Gorgoglione, Luca Proietti

**Affiliations:** 1 Department of Aging, Orthopaedic and Rheumatological Sciences, Fondazione Policlinico Universitario Agostino Gemelli IRCCS, 00168 Rome, Italy; scaramuzzolaura@gmail.com (L.S.); mariailaria.borruto01@icatt.it (M.I.B.); luca.proietti@policlinicogemelli.it (L.P.); 2 Department of Orthopaedics and Traumatology, Fondazione Casa Sollievo della Sofferenza IRCCS, 71013 San Giovanni Rotondo, Italy; perna.andrea90@gmail.com (A.P.); f.gorgoglione@operapadrepio.it (F.L.G.)

**Keywords:** minimally invasive, spinal metastasis, surgical management, spinal instability neoplastic, metastatic spinal disease, oncology, minimally invasive spine surgery, cancer surgery

## Abstract

**Background:** Managing vertebral metastases (VM) is still challenging in oncology, necessitating the use of effective surgical strategies to preserve patient quality of life (QoL). Traditional open posterior fusion (OPF) and percutaneous osteosynthesis (PO) are well-documented approaches, but their comparative efficacy remains debated. **Methods:** This retrospective study compared short-term outcomes (6–12 months) between OPF and PO in 78 cancer patients with spinal metastases. This comprehensive evaluation included functional, clinical, and radiographic parameters. Statistical analysis utilized PRISM software (version 10), with significance set at *p* < 0.05. **Results:** PO demonstrated advantages over OPF, including shorter surgical durations, reduced blood loss, and hospital stay, along with lower perioperative complication rates. Patient quality of life and functional outcomes favored PO, particularly at the 6-month mark. The mortality rates at one year were significantly lower in the PO group. **Conclusions:** Minimally invasive techniques offer promising benefits in VM management, optimizing patient outcomes and QoL. Despite limitations, this study advocates for the adoption of minimally invasive approaches to enhance the care of multi-metastatic patients with symptomatic VM.

## 1. Introduction

Bone constitutes one of the most common sites of metastasis in patients with advanced solid cancer, and the spine stands out as the primary location for osseous metastases [1]. Vertebral metastases (VMs), indicative of an advanced disease state, are significant contributors to disability, pain, and deterioration in the quality of life (QoL) among patients affected by metastatic solid tumors [2]. It is estimated that approximately 14% of patients with metastatic cancer exhibit symptomatic vertebral metastases (VMs); in 10% of patients, it represents the onset symptom of the disease [3]. Due to advancements in technologies and oncological treatments, coupled with the increased lifespan of cancer patients, this estimate is on the rise [3]. About 90% of patients with VMs have back pain, which is often followed by radicular pain [4,5]. The management of spinal metastases poses an escalating challenge for medical professionals and a multidisciplinary approach is needed [6]. Guiding this multidisciplinary approach is the Neurologic, Oncologic, Mechanical, and Systemic (NOMS) decision framework, which has become the standard in addressing the complexities of treatment [7]. In accordance with NOMS recommendations, surgical intervention is reserved for instances of neurological involvement (such as myelopathy or significant epidural spinal cord compression), the failure of radio/chemotherapy, or the presence of mechanical instability, which is established using the Spine Instability Neoplastic Score (SINS) [8]. While traditional open posterior fusion, with or without decompression, demonstrates efficacy in improving neurological status, its association with a high incidence of peri- and postoperative complications raises concerns about the ultimate outcomes and their subsequent impacts on patients’ QoL [9]. The advantages of percutaneous pedicle screw fixation, as evidenced by reduced blood loss, minimized soft tissue trauma, decreased perioperative pain, shorter hospitalization, and a quicker return to normal activities, have been extensively documented in polytrauma patients and those with degenerative spinal diseases [10]. Notably, the application of minimally invasive spinal surgery for spinal metastases has only gained traction in recent years. Several attempts to standardize treatment were undertaken during recent years, beginning with Gasbarrini et al.’s algorithm [11]; however, the present literature lacks conclusive evidence with which to establish the absolute superiority of one treatment modality over the other. Despite the absence of clear superiority, the use of percutaneous screw fixation has increased over the years, with encouraging clinical and biomechanical results. Considering all these aspects, the aim of this study is to compare short-term (6–12 months) functional, clinical, and radiographic outcomes in cancer patients with spinal metastases, anticipating a life expectancy of six months or more, who undergo percutaneous transpedicle osteosynthesis (PO) versus traditional open posterior fusion (OPF).

## 2. Materials and Methods

### 2.1. Research Design and Institutional Repository

This investigation constitutes a retrospective observational study, conducted within a singular medical center. We focused on cancer patients presenting with symptomatic vertebral metastases of solid tumors, devoid of neurological deficits. The cohort under scrutiny underwent evaluation and received care at our facility during a period spanning from January 2015 to December 2022. This study adhered to the guidelines outlined in the Strengthening the Reporting of Observational Studies in Epidemiology (STROBE) framework [12]. All participants in this research granted consent for the acquisition of scientific data while ensuring the utmost privacy. The collected data underwent anonymization processes to safeguard patient confidentiality and prevent identification. Given the study’s retrospective nature and the standard-of-care status of the procedures in our institution, combined with the comprehensive anonymity measures adopted for data collection, formal ethical committee approval was considered unnecessary. The study adhered to the principles outlined in the 1964 Declaration of Helsinki and its subsequent amendments.

During hospitalization for surgical treatment and the review of outpatient visit records for each participant, the following demographic parameters were collected: age, gender, comorbidities, and Body Mass Index (BMI). Pain assessment parameters were evaluated using the Numerical Rating Scale (NRS), while functional evaluation was conducted utilizing the Oswestry disability index (ODI) questionnaire. The identification of the primary tumor type; the assessment of overall health status, as measured by the Karnofsky Performance Score (KPS); and quality-of-life (QoL) appraisals were performed using the European Organization for Research and Treatment of Cancer QOL questionnaire, specially tailored for the bone metastases module (EORTC QLQ-BM22) [13]. Upon admission, the SINS was assessed for all patients.

### 2.2. Enrollment, Inclusion, and Exclusion Criteria

**Figure 1:**
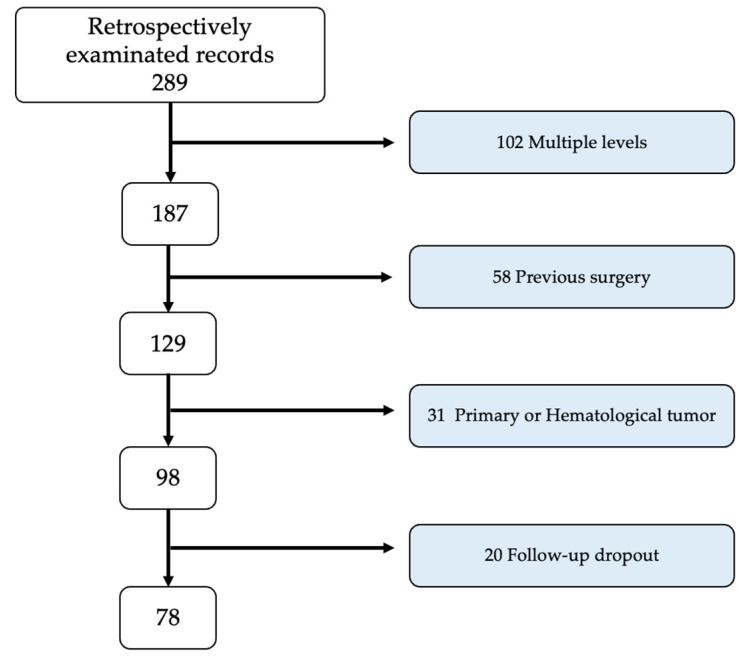
Flowchart of the patient selection process.

All patients accessing symptomatic vertebral metastases at our institution within the designated time frame were regarded as potential candidates for this study. The inclusion criteria encompassed the presence of a singular vertebral metastasis, a SINS score equal to or surpassing 7, a minimum follow-up period of 6 months, the comprehensive availability of clinical and radiographic records, an age above 18 years, and a life expectancy surpassing 6 months. The criteria for exclusion involved primary spinal tumors, prior spinal interventions, hematopoietic system tumors (myelomas, lymphomas), and the manifestation of neurological deficits. Patients who required decompression were excluded from this study to allow us to focus on cases that were suitable for stabilization procedures alone. Patients were categorized into two groups based on the received treatment: the open posterior fusion (OPF) group and the percutaneous osteosynthesis (PO) group. As this was a retrospective study, no randomization or specific choice of treatment type was performed. The criterion was purely temporal and dictated by the availability of the specific instruments and the radiolucent table in the operating room. Essentially, until 2018, we treated almost all patients with the OPF technique, while from 2019, we began using PO, resorting to the OPF technique only in cases where the radiolucent table or the necessary instruments were unavailable. A total of 289 patients were initially evaluated for inclusion in the study. The process of inclusion and exclusion is detailed in Figure 1. Briefly, patients were excluded for having multiple levels of disease (*n* = 102), previous surgeries (*n* = 58), primary tumors (*n* = 31), and follow-up dropout (*n* = 20). This resulted in a final cohort of 78 patients being included in the analysis. However, in Table 1 and Table 2, which concern the clinical data of the patients before dropout, the data of the entire cohort of 98 patients are reported; the statistical analysis of clinical and functional outcomes instead only considers the data of the 78 patients who were followed up.

### 2.3. Surgical Technique

All surgical procedures were performed by a team comprising two seasoned orthopedic surgeons specializing in spinal surgery. Prior to surgery, all patients received intravenous prophylactic antibiotic therapy with 2 g of cefazolin, administered approximately 30 min before incision. Surgery was carried out under general anesthesia with patients placed in the prone position on specialized spinal supports, utilizing a radiolucent carbon fiber operating table.

**Figure 2:**
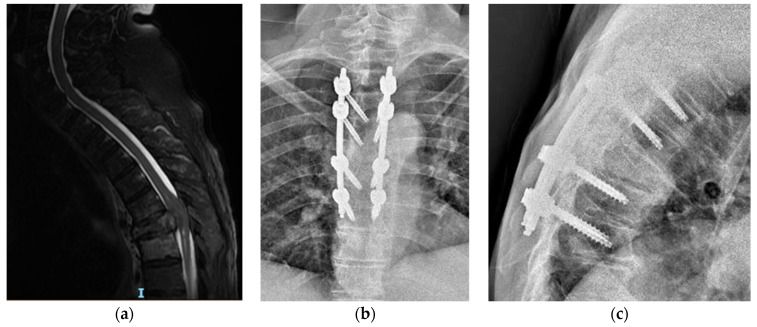
Case of open screw fixation. A 53-year-old female was accepted by our ER because of dorsal pain without any trauma. In her medical history, she recorded breast carcinoma, having been diagnosed 2 years ago and treated with surgery and chemotherapy. She missed regular follow-up appointments. In MRI scan images, we see (**a**) a lesion involving posterior elements (SINS 13), but without any cord compression. The patient underwent posterior open arthrodesis in order to stabilize the lesion (**b**,**c**).

For the OPF, the median posterior approach was employed, extending 2 levels above and below the affected segment. After skeletonizing the vertebral arches and exposing anatomical landmarks (facet joints, transverse processes, and isthmus), pedicle screws were placed using a freehand technique. Regular checks were performed with a standard C-arm fluoroscopy to ensure accurate screw placement. Vertebral stabilization was accomplished with two appropriately contoured titanium rods. Following thorough irrigation and hemostasis control, a layered suture was performed. An illustrative case of a patient treated with this technique is shown in Figure 2.

**Figure 3:**
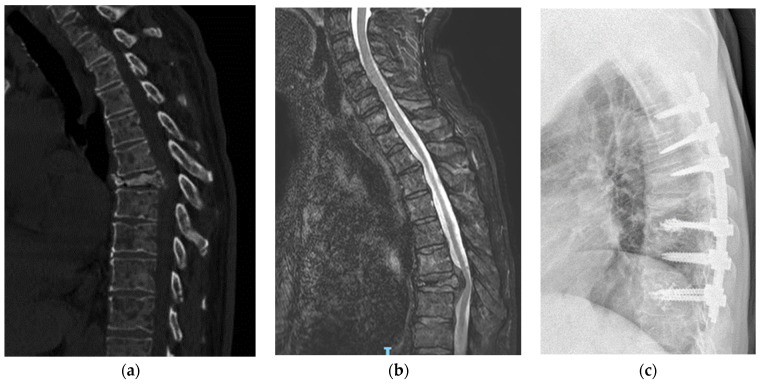
Case of percutaneous stabilization. A 83-year-old male, affected by hepatocarcinoma for 4 years, undergoing chemotherapy. During regular follow-up, CT scan image (**a**) showed T7 fracture. The patient presents other non-spinal multiple osteolytic lesions. In MRI image (**b**), mild compression is found. The patient was asymptomatic except for dorsal pain. Further analysis showed multiple lung and bone metastases. According to clinical symptoms, clinical condition and prognosis of the patients, we performed percutaneous screw fixation with augmented screws (**c**).

For the PO, the procedure began with the percutaneous placement of pedicle screws, using standard C-arm fluoroscopy to obtain anteroposterior and lateral projections. A four-handed technique was employed for surgery. Small incisions were made over the pedicles under fluoroscopic guidance. The entry point was then identified, and a trocar was inserted. Pedicle screws were inserted percutaneously using guide wires. Vertebral stabilization was completed with two titanium rods, which were appropriately contoured and inserted using specialized instruments. A layered closure was performed. An illustrative case of a patient treated with this technique is reported in Figure 3.

During surgery for both techniques (PO and OPF), the bone quality was assessed, and based on the screw stability, augmentation with polymethylmethacrylate (PMMA) of the screws was necessary in some cases.

In all patients, a transpeduncular biopsy was performed, using dedicated trocars at the site of the lesion to confirm the diagnosis. This procedure involved the meticulous extraction of tissue samples for diagnostic validation.

### 2.4. Clinical and Radiological Evaluation

Patients underwent preoperative clinical assessments and were followed up at 1 month, 6 months, and 1 year post-surgery. Subsequent evaluations, incorporating the ODI questionnaire and the NRS for back pain, were carried out during each follow-up visit. The patient’s general health status was appraised using the KPS and the need for surgical intervention SINS in all patients. QoL was systematically evaluated through the EORTC QLQ-BM22 before surgery, at 6 months, and at one year post-surgery. The EORTC QLQ-BM22 is a pivotal 22-item questionnaire specifically designed for the palliative cancer population experiencing bone metastases. This module serves as a cornerstone for measuring the quality of life (QoL) in cancer clinical trials. Widely adopted, it stands as the primary tool for QoL assessment in bone metastases patients in both Europe and Canada [14].

All study participants underwent comprehensive radiological assessments. Preoperatively, each patient received both spinal CT and MRI scans, along with anteroposterior and lateral X-rays. Postoperatively, radiographic evaluations primarily relied on spine X-rays at 1, 6, and 12 months, with CT and MRI reserved for specific cases or patients encountering complications. The objective of the radiographic follow-up was to monitor hardware integrity and assess the stability of the construct over time. The incidence of complications, including surgical site infections, urinary tract infections, pneumonia, new neurologic deficits, blood transfusions, and strokes, was reported.

### 2.5. Peri and Postoperative Follow Up

The perioperative information that was collected and analyzed consisted of the following: operative duration, number of instrumented segments, blood loss, the requirement for intensive care unit (ICU) admission, and length of hospital stay. Perioperative blood loss was quantified by determining the volume in suction canisters, with the saline lavage volume subtracted. Additionally, the requirements for postoperative transfusions and hemoglobin levels were assessed both preoperatively and on the second postoperative day. Transfusion was exclusively reserved for patients exhibiting hemoglobin levels below 8 g/dL.

All patients were assisted into a seated position on the first day postoperatively. For patients undergoing OPF, removal via surgical drainage occurred on the second day after the operation. The urinary catheter was typically removed on the second postoperative day for all patients. Postoperatively, patients were mobilized into an upright position on the second day, utilizing a reinforced canvas brace worn for the subsequent three months during seated and upright positions. Every participant underwent prophylactic antithrombotic, therapy utilizing low-molecular-weight heparin for the subsequent 30 days post-surgery. Patients who required postoperative radiation followed a standardized protocol, with radiation typically administered within 4–6 weeks post-surgery.

### 2.6. Variables

The primary outcomes of this study involve assessing quality of life (QoL) through the EORTC QLQ-BM22, with clinical and functional evaluations conducted using the NRS scale and ODI. Secondary outcomes encompass the count of perioperative complications, mortality at 6 and 12 months, the incidence of hardware breakages, and any delayed complications.

### 2.7. Statistical Analysis

The statistical analysis was conducted using dedicated software, specifically PRISM from GraphPad Software (version 10). Data are expressed as means and percentages, rounded to one decimal place. The standard deviation (SD) for each calculated value was reported. The Mann–Whitney U-test was applied for the analysis of independent ordinal variables, while the Wilcoxon Signed-Rank Test was used for the analysis of two dependent ordinal variables. The Chi-square test was employed to assess categorical data. The normality of the study population was assessed using the Shapiro–Wilk test. Since the population did not display a normal distribution, ANOVA was deemed unsuitable and, consequently, not performed. A *p* value < 0.05 was considered statistically significant.

## 3. Results

### 3.1. Patients

During the retrospective analysis of 289 patients, a mere 98 aligned with the predetermined inclusion and exclusion criteria. Strikingly, a subset of only 78 individuals demonstrated compliance by successfully fulfilling the 6-month follow-up requirements, warranting their inclusion in the study. Patients were divided into the OPF (originally 58, then 42) and PO (originally 40, then 36) groups based on their treatment regimen. The average age of the population sample was 68.3 (±13.8), with a male-to-female ratio of 39 to 59. The mean KPS before the surgery was 59.3 (±8.2). The SINS was below 13 in 58.8% of patients and was above 13 in 41.2%. No significant demographic differences were observed between the two groups. Table 1 provides a summary of the main characteristics of the study population.

### 3.2. Surgical Data

The average surgical procedure duration was 91 min shorter in the PO group compared to the OPF group, and this difference was found to be statistically significant (*p* = 0.035). Blood losses were also significantly reduced in the PO group, with a decrease of approximately 418 mL per procedure (*p* = 0.029) compared to the OPF group. This finding was reinforced by the fact that postoperative hemoglobin levels in the OPF group were, on average, 2 g/dL lower than those in the PO group, resulting in a higher incidence of homologous blood transfusions in the OPF group (41.3% vs. 12.5%, *p* = 0.031). The average hospital stay was nearly 5 days shorter in the PO group, and this difference was statistically significant. Patients in the OPF group necessitated a higher rate of postoperative admission to the intensive care unit (ICU) compared to those in the PO group, and this difference proved statistically significant (OPF 29.2% vs. PO 7.5%, *p* = 0.021).

Every patient underwent a transpeduncular biopsy, confirming the initial diagnosis. However, a noteworthy exception arose in a male participant from the OPF group, presenting with lung carcinoma. Surprisingly, the biopsy uncovered metastases that stemmed from prostate carcinoma, challenging the initial assessment. The key surgical data are outlined in Table 2.

### 3.3. QoL and Clinical Outcomes

The measured improvement in QoL, as determined based on the QLQ-BM22 score, displayed a notable elevation in both the functional and symptom scales for both patient groups following surgical intervention. This positive trend persisted consistently at the 6- and 12-month follow-ups for those subjects who completed the entire follow-up process, demonstrating statistical significance. A comparative analysis of the OPF and PO groups unveiled a notably greater enhancement in the quality of life within the latter, which was particularly evident in the symptom scales at the 6-month mark, with this distinction achieving statistical significance. Despite the PO group presenting superior values to the OPF group, these disparities did not attain statistical significance. In the evaluation of pain using the NRS scale, a considerable reduction was observed in both groups post-surgery, and this amelioration remained steady at the 6- and 12-month follow-ups. The PO group exhibited lower and more statistically significant values than the OPF group at 6 and 12 months post-surgery (PO 3.7 ± 1.4 vs. OPF 6.1 ± 2.2, *p* = 0.038; PO 3.1 ± 0.9 vs. OPF 4.9 ± 1.9, *p* = 0.027). Furthermore, patient functional status, assessed using the ODI scale, notably improved for all patients post-surgery. However, the sole statistically significant difference between the two groups was identified at the 6-month follow-up (PO 18.8 ± 9.8 vs. OPF 37.1 ± 8.8, *p* = 0.043). The key data are reported in Table 3.

### 3.4. One-Year Mortality and Complications

The one-year mortality rate within the entire study cohort was recorded at 14%. However, upon closer examination of the two cohorts, a noteworthy distinction emerged: OPF patients displayed a one-year mortality rate of 21.4%, as opposed to the 5.5% observed in the PO group. This discrepancy proved statistically significant (*p* = 0.041). In terms of complications, they manifest more prevalently within the OPF group, encompassing instances of both superficial and deep infections, along with occurrences of pulmonary thromboembolism. Remarkably, the singular case of screw loosening, managed conservatively with the patient reporting no pain, was exclusively observed within the PO group. A summary of complications recorded is reported in Table 4.

### 3.5. Subgroup Analysis

An important issue to evaluate regarding complications, clinical outcomes, and mortality rates concerns the need for adjuvant radiotherapy in patients with spinal metastasis. In our cohort, only 20 patients underwent pre-op radiotherapy as they presented radiosensitive histotypes. Specifically, 13 patients had breast carcinoma and 7 patients had prostate carcinoma. Of these, 10 belonged to the OPF Group (7 breast and 3 prostate) and 10 to the PO Group (6 breast and 4 prostate). Comparing the complication rates, we noted that 3 out of the 5 surgical wound infections in the OPF Group occurred in patients who underwent radiotherapy, with a rate of 50%, while none of the patients in the PO Group who underwent radiotherapy reported surgical wound infections (these data are statistically significant, *p* = 0.0023). Regarding reported mechanical complications, specifically screw loosening, the only patient in the PO Group who reported this complication did not belong to the group that underwent radiotherapy. There are no statistically significant differences in mortality between the two subgroups.

## 4. Discussion

### 4.1. Background and Findings

There is growing interest in the management of vertebral metastases (VMs) in contemporary times, fueled by their significant societal impact and the consequential economic implications for healthcare systems [9]. The spine stands as the predominant site for the dissemination of bone metastases, contributing to roughly 50% of all instances of secondary malignant growths [5,9]. VMs significantly and frequently exacerbate the already compromised QoL in these patients, primarily due to unrelenting, intractable pain and pathological fractures resulting from the oncological disease. Our study found that the PO group demonstrated a significantly shorter surgical duration, reduced blood loss, and shorter hospital stays compared to the OPF group. Additionally, the PO group had lower perioperative complication rates and a lower one-year mortality rate. These findings suggest that minimally invasive techniques may offer substantial benefits for certain patient populations.

The recent surge in the VM incidence is attributed to advancements in the treatment and diagnosis of oncological pathologies, which have extended the lifespan of patients in the terminal stage of this disease [15,16]. Recognizing this, the standardization of the treatment of these lesions becomes imperative, with the primary goal of preventing undesired sequelae and preserving, or optimally enhancing, the quality of the remaining life [16]. Increasingly, surgery for VMs does not pursue a curative intent; rather, the surgical objectives primarily involve providing mechanical stability to the lesions, alleviating pain via its reduction to acceptable levels, and preventing neurological sequelae [5,17]. Several aggressive surgical strategies have been devised to enhance outcomes for patients grappling with metastatic spine disease. However, these strategies carry a high burden of morbidity and complication rates, especially in patients that are burdened with numerous neoplasm-related comorbidities [5,9]. As a result, the indication for aggressive surgery is very limited, if not prohibited, in patients with poor overall health and reduced life expectancy, as is evident in our series. Undoubtedly, surgery occupies a central position in the therapeutic landscape of VMs, with the recommended approach being contingent upon variables such as symptomatology, neurological involvement, radiotherapy sensitivity, overall patient health, tumor histology, and disease stage. Decision making often relies on diverse scoring systems, including the NOMS framework [7,18] and SINS score [8]. Evolution in spinal surgery, particularly the integration of less invasive methodologies, has markedly improved clinical outcomes across various pathologies. This paradigm shift has demonstrated significant promise in VM treatment, as underscored in a recent meta-analysis [9]. The findings revealed reduced blood loss; improved clinical outcomes, linked to expedited scar recovery; and the judicious integration of radiotherapy when deemed necessary. Consequently, this transformative approach resulted in substantially enhanced clinical outcomes for VM patients. As a consequence, the incorporation of minimally invasive spine surgery (MISS) has become pivotal in the management of spinal metastases, providing a standardized treatment modality. Adhering to the indications published in the 2012 Oncological Guidelines for Spinal Metastases Management, the embrace of minimally invasive techniques has ushered in noteworthy advancements in this domain [19]. This methodology is characterized by a lower complication rate, abbreviated hospitalization durations, and expedited postoperative recuperation. The merits inherent in these methodologies encompass minimized incision sizes, thereby mitigating the complications related to wound healing. Moreover, these techniques eliminate the need for detaching and retracting back muscles, averting postoperative pain and excessive bleeding. Consequently, this leads to a reduction in intraoperative blood loss, obviating the necessity for supplemental blood transfusions. The critical significance of these advantages lies in their role in upholding and enhancing the QoL of cancer patients with limited life expectancy, be it in the short or medium term [5,9,20,21].

Our cohort is composed of 78 patients with diverse primary neoplasms. Characterized by a low KPS score and vertebral pathological or impending fractures that affect QoL, all patients underwent fixation. This intervention was prompted by clear preoperative instability or was used as a preventative measure against instability (SINS score greater than or equal to 7) [8]. None of the patients in the series were indicated for a major surgical approach aiming for gross-total or complete resection of metastases. We used the shortest feasible implant in all patients in our series, considering the lesion’s biological behavior (osteolytic, osteosclerotic or mixed) and the number of involved segments.

The MISS technique, designed to evade extensive exposure and detachment of paraspinal muscles through the percutaneous insertion of pedicle screws and rods, demonstrated advantages over standard open fixation methods [22]. Our results highlighted the significant advantages of MISS over standard open techniques, including reduced blood loss, shorter operation duration, and decreased hospital stay. Safety was underscored by the lower peri- and postoperative complication rate in the PO group.

A significant finding highlighted in our study is the lower incidence of superficial infections in the PO group compared to the OPF group. Specifically, these complications were much more prevalent in patients who underwent pre- and postoperative radiotherapy and were treated with the OPF technique, as reported in the subgroup analysis. 

The perceived advantage of MISS in swiftly enhancing QoL, particularly in the immediate postoperative phase, aligned with its effectiveness in reducing postoperative pain related to both surgery and spinal metastasis. However, these distinctions between the two groups diminished after a 12-month follow-up. This was mirrored in the evaluation of the functional score (ODI), as reported in Table 3.

Few authors evaluate implant-related complications when discussing the pathological fractures caused by spinal metastases. In our series, only one case of screw loosening was observed in the PO group patients; this did not require reintervention as the patient remained asymptomatic for pain. No instances of pullout or implant breakage were noted. These outcomes may have been influenced by the relatively short follow-up period; regrettably, an extended follow-up was potentially influenced by the progression of the underlying disease, particularly given the fact that the majority of patients had a life expectancy just slightly surpassing one year. This potential bias is an important factor to consider in evaluating the long-term outcomes. Our analysis of the differences in one-year mortality rates between the OPF and PO groups revealed significant variations. We considered the potential biases introduced by the exclusion of certain patients from the study. It appears that the notable difference in one-year mortality rates is not solely attributable to perioperative complications. Instead, this discrepancy may be influenced by several factors, including patient selection criteria and underlying health conditions. These findings highlight the importance of considering broader patient characteristics and pre-existing conditions when evaluating surgical outcomes in spinal metastasis treatments.

Our findings aligned with those of previous studies that suggested minimally invasive spine surgery (MISS) offered significant advantages over traditional open procedures. For example, Meyer et al. (2023) reported a substantial reduction in surgical site infections with MISS compared to open surgery in a large cohort of patients undergoing lumbar decompression and fusion [23]. Similarly, Conti et al. (2019) emphasized the improved quality of life and reduced perioperative morbidity associated with MISS in patients with metastatic spinal cord compression [24]. Another study by Pennington et al. (2018) reviewed the historical and modern developments of minimally invasive techniques, noting their efficacy in reducing operative times and enhancing recovery [25]. These studies, along with our own, underscore the potential benefits of adopting MISS as a standard approach for managing spinal metastases in appropriately selected patients.

An additional advantage of MISS lies in its increasingly frequent integration with navigation techniques and robot-assisted surgery. The implementation of these innovative techniques is crucial, even in the case of multi-metastatic patients, as it aligns with the evolving paradigm of personalized and patient-based approaches sought in modern medicine.

### 4.2. Limitations

The current study is not without limitations. Firstly, the sample size is relatively small; however, this is inherent to the specificity of the topic. Secondly, the study has a retrospective nature. Thirdly, the relatively brief follow-up period is dictated by the primary pathology and the short life expectancy of the patients. Finally, a notable limitation of our study is the exclusion of patients requiring decompression. Consequently, our findings may not be applicable to cases that necessitate decompression, and future studies should aim to include such cases to provide a more comprehensive evaluation of surgical outcomes for all patients with spinal metastases.

## 5. Conclusions

Posterior stabilization emerges as an indispensable yet palliative measure for multi-metastatic cancer patients grappling with symptomatic VMs without neurological compression. This method is designed to safeguard and, when feasible, improve QoL. While the safety and subsequent improvement in functional and clinical outcomes at one year post-surgery remain comparable between MISS and conventional open procedures, MISS presents distinct advantages. Notably, it demonstrates a reduction in surgical duration, minimized blood loss, abbreviated hospital stays, and a diminished reliance on both blood transfusions and postoperative ICU admission. This comprehensive refinement results in a tangible enhancement of QoL and functional metrics, particularly within the initial six months following the intervention.

From our perspective, the pioneering choice for spinal metastatic patients without neurological status alteration should unequivocally favor MISS techniques. These techniques offer a level of spine fixation comparable to that of traditional open surgical approaches in terms of safety and effectiveness in short-term follow-ups, but also strategically alleviate the procedural impact on patients in critical conditions.

## Figures and Tables

**Table 1:** Demographic features. BMI: Body Mass Index; KPS: Karnofsky Performance Score; F: female; M: male; OPF: open posterior fusion; PO: percutaneous osteosynthesis; SINS: spine instability neoplastic score; * OPF vs. PO.

Demographics	Group OPF	Group PO	Total	*p* Value *
*n*° of patients	58	40	98	
Age	68.7 (±13.5)	67.7 (±14.1)	68.3 (±13.8)	0.877
Sex ratio (M/F)	23:35	16:24	39:59	
BMI	27.6 (±3.2)	26.9 (±2.9)	27.2 (±3.1)	0.862
KPS	59.7 (±7.1)	58.5 (±9.1)	59.3 (±8.2)	0.654
SINS				
Between 7 and 12	35 (60.3%)	25 (62.5%)	60 (58.8%)	0.873
Between 13 and 18	23 (39.6%)	15 (37.5%)	38 (41.2%)	0.766
2 o comorbidities	34 (58.6%)	29 (72.5%)	64 (62.7%)	0.834
Type cancer				
Lung	23 (39.6%)	17 (42.5%)	40 (39.2%)	0.634
Breast	7 (12%)	6 (15%)	13 (12.7%)	0.787
Prostate	3 (5.2%)	4 (10%)	7 (6.9%)	0.689
Kidney	7 (12%)	6 (15%)	13 (12.7%)	0.801
Ovary	6 (10.1%)	4 (10%)	10 (9.8%)	0.742
Thyroid	5 (8.6%)	1 (2.5%)	6 (5.9%)	0.037
Other	7 (12%)	2 (5%)	9 (8.8%)	0.692
Type of metastasis				
Osteolytic	39 (67.1%)	28 (70%)	67 (65.7%)	0.894
Osteosclerotic	16 (27.5%)	8 (20%)	32 (31.4%)	0.761
Mixed	3 (5.2%)	4 (10%)	7 (6.9%)	0.882

**Table 2:** Surgical data. Hb: hemoglobin; ICU: intensive care unit; Los: length of stay. OPF: open posterior fusion; PO: percutaneous osteosynthesis.

Surgical Parameters	Group OPF	Group PO	*p* Value
*n*° of patients	58	40	
Operative time	192 (±49) min	101 (±27) min	0.035
Estimated intraoperative blood loss	521.5 (±186) mL	103.4 (±54) mL	0.029
Preoperative Hb value	11.1 (±2.1) g/dL	11.4 (±1.8) g/dL	0.398
Postoperative Hb value	7.9 (±1.7) g/dL	10.9 (±1.2) g/dL	0.038
Patients requiring blood transfusion	24 (41.3%)	5 (12.5%)	0.031
Patients requiring ICU	17 (29.2%)	3 (7.5%)	0.021
Los (days)	9.7 (±2.4)	5.0 (±1.2)	0.037
Instrumented Level			
3	16 (27.5%)	8 (20%%)	0.897
5	34 (41.3%)	25 (62.5%)	0.832
7	8 (13.8%)	7 (17.5%)	0.617

**Table 3:** Quality of life (QoL) and main clinical and functional outcomes. FS: functional scale; NRS: numeric rate scale; ODI: Oswestry disability index; OPF: open posterior fusion; PO: percutaneous osteosynthesis; QLQ-BM22: bone metastases module; SS: symptom scale. Regarding the QLQ-BM22 functional scales, the scores span from 0 to 100, with higher scores indicating an improved quality of life. As for the QLQ-BM22 symptom scales, the scores also range from 0 to 100, but higher scores signify more severe symptoms.

	Preoperative	6 Months	1 y	*p* Value *, ** Value Compared
QLQ-BM22 FS, OPF	56.7 (±9.5) *	71.2 (±7.4)	72.7 (±8.2) **	0.029
QLQ-BM22 FS, PO	57.5 (±10.5) *	72.7 (±8.3)	74.2 (±6.9) **	0.031
*p* Value OPF vs. PO	>0.05	>0.05	>0.05	
QLQ-BM22 SS, OPF	24.2 (±4.2) *	18.2 (±2.1)	16.7 (±3.4) **	0.048
QLQ-BM22 SS, PO	23.8 (±5.3) *	11.6 (±3.2)	10.5 (±2.9) **	0.032
*p* Value OPF vs. PO	>0.05	0.035	>0.05	
NRS Group OPF	9.1 (±3.2) *	6.1 (±2.2)	4.9 (±1.9) **	0.044
NRS Group PO	9.3 (±2.9) *	3.7 (±1.4)	3.1 (±0.9) **	0.012
*p* Value OPF vs. PO	>0.05	0.038	0.027	
ODI Group OPF	61.4 (±10.8) *	37.1 (±8.8)	27.7 (±7.2) **	0.037
ODI Group PO	63.4 (±9.9) *	18.8 (±9.8)	21.3 (±9.1) **	0.021
*p* Value OPF vs. PO	>0.05	0.043	>0.05	

**Table 4:** Complications and 1-year mortality. OPF: open posterior fusion; PO: percutaneous osteosynthesis; * OPF vs. PO.

Complications	Group OPF	Group PO	Total	*p* Value *
*n*° of patients	42	36	78	
Wound infection	5(11.9%)	1 (2.8%)	6 (7.8%)	0.024
Deep infection	2 (4.8%)	0	2 (2.6%)	0.037
Thromboembolism	2 (4.8%)	0	2 (2.6%)	0.031
Screw loosening	0	1 (2.8%)	1 (1.3%)	0.044
Hardware failure	0	0	0	-
Tardive neurological deficit	2 (4.8%)	1 (2.8%)	3 (3.8%)	>0.05
1-year mortality	9 (21.4%)	2 (5.5%)	11 (14%)	0.041

## Data Availability

Data are available by request to the author.
